# Multiple lifestyle behaviour mHealth intervention targeting Swedish college and university students: protocol for the *Buddy* randomised factorial trial

**DOI:** 10.1136/bmjopen-2021-051044

**Published:** 2021-12-30

**Authors:** Katarina Åsberg, Oskar Lundgren, Hanna Henriksson, Pontus Henriksson, Preben Bendtsen, Marie Löf, Marcus Bendtsen

**Affiliations:** 1 Department of Health, Medicine and Caring Sciences, Linköping University, Linköping, Sweden; 2 Department of Medical Specialist, Motala Hospital, Motala, Sweden; 3 Department of Biosciences and Nutrition, Karolinska Institutet, Stockholm, Sweden

**Keywords:** public health, preventive medicine, epidemiology

## Abstract

**Introduction:**

The time during which many attend college or university is an important period for developing health behaviours, with potentially major implications for future health. Therefore, it is concerning that many Swedish students excessively consume alcohol, have unhealthy diets, are not physical active and smoke. The potential of digital interventions which integrate support for change of all of these behaviours is largely unexplored, as are the dismantled effects of the individual components that make up digital lifestyle behaviour interventions.

**Methods and analysis:**

A factorial randomised trial (six factors with two levels each) will be employed to estimate the effects of the components of a novel mHealth multiple lifestyle intervention on alcohol consumption, diet, physical activity and smoking among Swedish college and university students. A Bayesian group sequential design will be employed to periodically make decisions to continue or stop recruitment, with simulations suggesting that between 1500 and 2500 participants will be required. Multilevel regression models will be used to analyse behavioural outcomes collected at 2 and 4 months postrandomisation.

**Ethics and dissemination:**

The study was approved by the Swedish Ethical Review Authority on 2020-12-15 (Dnr 2020-05496). The main concern is the opportunity cost if the intervention is found to only have small effects. However, considering the lack of a generally available evidence-based multiple lifestyle behaviour support to university and college students, this risk was deemed acceptable given the potential benefits from the study.

Recruitment will begin in March 2021, and it is expected that recruitment will last no more than 24 months. A final data set will, therefore, be available in July 2023, and findings will be reported no later than December 2023.

**Trial registration number:**

ISRCTN23310640; Pre-results.

**Strengths and limitations of this study**

- A factorial trial is used to estimate the effects of the components of a novel mHealth intervention on four important health-related behaviours: alcohol consumption, diet, physical activity and smoking.
- Employing a Bayesian group sequential design will ensure that the trial will not be underpowered, nor recruit more participants than necessary.
- Self-reported outcomes are used, which may be vulnerable to bias from research participation effects, including the risk of detection bias from telephone follow-ups.
- Mediators are measured using single face-valid items rather than validated questionnaires in order to reduce participant burden.

## Introduction

Non-communicable diseases (NCDs), such as cardiovascular diseases and cancers, constitute a major public health concern by causing 71% of deaths globally each year.^1^ The WHO has made it clear that the burden of disease that NCDs cause would be greatly reduced if the prevalence of harmful alcohol consumption, unhealthy diets, physical inactivity and smoking^2^ was reduced. Behavioural risk factors, such as an individual’s lifestyle, additionally accounted for 36% of all disability-adjusted life years in 2017 globally.^3^ Thus, it is important to find effective and scalable means of helping individuals to improve their lifestyle behaviours in order to improve health and well-being.

Many health-related behaviours are established during adolescence and young adulthood and frequently persist into adulthood.^4–6^ The time during which many attend college or university is an important period for developing healthy lifestyle behaviours, with potentially major implications for future health. Becoming a student is in Sweden often associated with several new commitments, such as caring for a household, building new social networks and dealing with basic household economics. It is, therefore, particularly concerning that many Swedish students have unhealthy lifestyle behaviours.^7 8^

While there is a lack of specific data regarding college and university students’ lifestyle behaviours in Sweden, data on the 18–29 age group from the Public Health Agency of Sweden’s national public health survey from 2018 (n=1925) suggest that the majority of these individuals (94%) report at least one risk behaviour: 17% were smokers (5.4% daily and 11.1% occasionally), 25% had a risky alcohol consumption, 28% were not sufficiently physically active, 92% did not eat enough fruit and vegetables and 32% were overweight or obese. Approximately 27% reported two or more risk behaviours, risky drinking and not eating enough fruit and vegetables being the most common. Consequently, it is important to empower young adults with the knowledge, attitudes and life skills necessary for making informed decisions that are protective of good health and can reduce the future risk of NCDs.^9^

### Digital interventions and mHealth

Young adults are digital natives having easy access to technology and commonly use apps and the internet to seek health information.^10^ Therefore, using digital devices represents a well-established means of delivery of personalised health interventions to young adults.^10–12^ Interventions which use mobile technologies, often referred to as *mHealth* interventions, are of particular interest as they offer new potential in delivering behaviour change support in individuals’ everyday life.

Several *single* behaviour digital lifestyle interventions have been evaluated among college and university students over the past decades,^13–16^ including various combinations of delivery modes, such as text messaging and web-based platforms.^17–24^ In Sweden for instance, research on digital interventions have shown promising results with respect to alcohol,^25–32^ smoking cessation^33 34^ and mental health promotion.^35^ However, few studies investigate interventions which aim to change two or more unhealthy lifestyles simultaneously.^36–41^ Thus, while the potential of digital behaviour interventions is promising, knowledge about the effects of digital *multiple* lifestyle interventions is limited—despite unhealthy behaviours tendency to co-occur.^42^

In addition to the evidence for digital multiple lifestyle interventions being limited, current evidence for behaviour interventions lacks detail with respect to the effects of the components of interventions. While there have been trials which aim to dismantle the effects of intervention components,^25 43 44^ most trials estimate the effects of interventions as a whole.^45^ Increasing our understanding of the effects at the component level, in particular with respect to multiple lifestyle behaviours, may help move the field of behaviour interventions forward.

### Aims and objectives

The aim of this study is to estimate the effects of the components of a novel mHealth intervention on multiple lifestyle behaviours (alcohol, diet, physical activity and smoking) among college and university students in Sweden. The study is a part of the Mobile health Multiple Behaviour Interventions across the LifEspan research programme (MoBILE),^46^ which contains seven projects on multiple lifestyle interventions among different populations across the lifespan. The objectives of the study are to:

1. Estimate the effects of a novel mHealth intervention’s different components on individual lifestyle behaviours:
  - Weekly alcohol consumption and number of episodes per month of heavy drinking.
  - Weekly consumption of sugary drinks and average daily fruit and vegetable consumption.
  - Weekly moderate to vigorous physical activity (MVPA).
  - Four-week point prevalence of smoking.
2. Estimate the degree to which the effects of the components are mediated through perceived importance, confidence and know-how.
3. Detect interactions among lifestyle behaviour change, for example, those who stop smoking may also reduce their alcohol consumption, and the degree to which this is moderated by the components of the intervention.

## Methods

A factorial randomised trial^47^ (six factors with two levels each) will be employed to address the objectives of the study. A Bayesian group sequential design will be employed to periodically make decisions to continue or stop recruitment.^48–50^ This protocol contains relevant items from the *Standard Protocol Items: Recommendations for Interventional Trials*.^51^

### Study setting, recruitment and eligibility

All 31 college and universities in Sweden will be invited to participate in the trial, and we anticipate that the majority will accept. Participating universities will recruit students to the trial using: (1) paper advertising (posters and leaflets), (2) digital advertising (email, website, social media) and (3) through student healthcare staff. Students will register their interest by sending a text message to a dedicated telephone number (included in all information materials). In response, students will receive a text message with a hyperlink to a web page presenting informed consent materials (online supplemental appendix A). All students who consent, by clicking on a button after reading the informed consent materials, will immediately be asked to complete an online baseline questionnaire (online supplemental appendix B), which will also be used to assess eligibility for the trial.

10.1136/bmjopen-2021-051044.supp1 Supplementary data

10.1136/bmjopen-2021-051044.supp2 Supplementary data

Students will be included in the trial if they fulfil at least one of six conditions:

- **Weekly alcohol consumption:** consumed 10/15 (female/male) or more standard drinks of alcohol the past week. A standard drink of alcohol is in Sweden defined as 12 g of pure alcohol.
- **Heavy episodic drinking:** consumed 4/5 (female/male) or more standard drinks of alcohol on a single occasion at least once the past month.
- **Fruit and vegetables:** consumed less than 500 g of fruit and vegetables on average per day the past week.
- **Sugary drinks:** consumed 3 or more units of sugary drinks the past week. One sugary drink unit is defined as approximately 33 cl.
- **MVPA:** spent less than 150 min on MVPA the past week.
- **Smoking:** having smoked at least one cigarette the past week.

Students will be explicitly excluded if they do not fulfil any of the criteria or if they are less than 18 years of age. The trial information and intervention will be entirely in Swedish and delivered to participants’ mobile phones, thus not comprehending Swedish well enough to sign up or not having access to a mobile phone will implicitly exclude participants.

### Interventions

The *Buddy* multiple lifestyle behaviour intervention is an mHealth intervention which consists of six components which users access using their mobile phone, based on an intervention design we have used previously.^52^ The intervention is designed around social cognitive theories of behaviour change, with a focus on modifying environment, intention, and skills.^53 54^ Please see online supplemental appendix C for full details. The intervention’s components are intended to be used as a toolbox, allowing users to choose which parts of the intervention to interact with and tailor the support to their needs. The intervention materials can be accessed at participants’ discretion over a 4-month period, and each Sunday afternoon participants will receive a text message with a link and a reminder to access *Buddy*.

10.1136/bmjopen-2021-051044.supp3 Supplementary data

The six components of the intervention are: (1) screening and feedback, (2) goalsetting and planning, (3) motivation, (4) skills and know-how, (5) mindfulness, and (6) self-authored text messages. These components will also represent factors in the factorial trial. Participants eligible for the trial will be randomly allocated to 1 of 64 factorial conditions, each condition representing a unique combination of *Buddy*’s six components—which are either present or absent (2^6^=64 conditions). They will remain in the same condition for the entirety of the 4-month intervention period. For a more detailed description of each component, including a full specification of each factorial condition, please see online supplemental appendix C.

### Outcomes

#### Measures

Outcomes are listed here and subsequently explained. All questionnaires (baseline, 1-month, 2-month and 4 month follow-up) used in the trial are found in online supplemental appendix B.

##### Primary outcome measures

- **Alcohol:** weekly alcohol consumption; monthly frequency of heavy episodic drinking.
- **Diet:** average daily consumption of fruit and vegetables; weekly consumption of sugary drinks.
- **Physical activity:** weekly MVPA.
- **Smoking:** 4-week point prevalence of smoking abstinence.

##### Secondary outcome measures

- Weekly consumption of candy and snacks.
- Body mass index (BMI).
- Number of cigarettes smoked the past week.
- Perceived stress.

##### Mediation measures

- Importance of change.
- Confidence in one’s ability to change.
- Knowledge of how to change.

#### Primary and secondary outcome measures

Weekly alcohol consumption will be assessed by asking participants the number of standard drinks of alcohol they consumed last week (short-term recall method^55^). Frequency of heavy episodic drinking will be assessed by asking participants how many times they have consumed more than 4/5 (female/male) standard drinks of alcohol on one occasion the past month. These two outcomes are both part of the proposed core outcome set for brief alcohol interventions.^56–58^

Diet and physical activity will be measured using a questionnaire based on the previously published questionnaire by the National Board of Health and Welfare in Sweden^59^ and was further modified to also include portion sizes. The consumption of fruit and vegetables will be measured using two questions concerning the number of portions (100 g) of fruit and vegetables (respectively) the participants ate on average per day during the past week. Sugary drink consumption will be measured by a question regarding the number of units (33 cl) of sugary drink participants consumed the past week. MVPA will be estimated by summing responses to two questions regarding the number of minutes spent on moderate and vigorous physical activity, respectively, during the past week.

BMI will be measured by asking participants to report their weight (both weight and height have been reported at baseline, and height is unlikely to have changed significantly and will, thus, only be asked at baseline).

Four-week point prevalence of smoking abstinence (no cigarettes the past week) will be asked as a binary question. This is a suggested measure by the Society of Research on Nicotine and Tobacco.^60^ Participants who have smoked any cigarette the past 4 weeks will be asked for the number of cigarettes smoked the past week.

Perceived stress will be assessed using the short form perceived stress scale.^61^ There may be a risk that participants experience an increased level of stress as they change their behaviours, for instance, many may have used smoking as a destressor. Findings in relation to this outcome may be useful to address such hypotheses in future research.

#### Mediation measures

To further understand how the interventions’ components may affect behaviour change, participants will be asked to report on psychosocial factors believed to be important markers of behaviour change.^53 54 62–64^ Confidence, importance and know-how will be measured using single face-valid items (see online supplemental appendix B), a limitation which we point out in the discussion but which we find necessary in order to reduce participant burden. These measures will be used to estimate to which degree the total effects of the components of the intervention are mediated through these factors.

### Participant timeline and follow-ups

**Figure 1:**
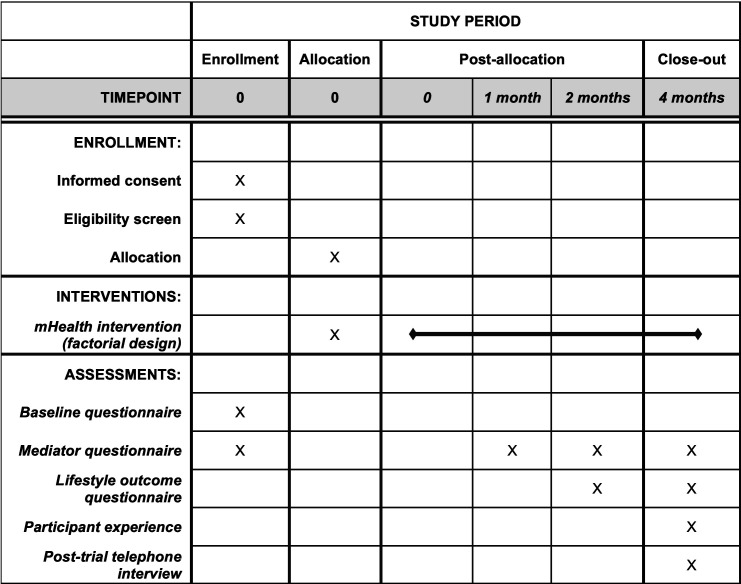
SPIRIT figure depicting participant timeline. SPIRIT, Standard Protocol Items: Recommendations for Interventional Trial.

A trial participant timeline is presented in figure 1. Intervention components (depending on allocation) will be made available to participants all at once and stay available to participants at their own discretion throughout the 4-month trial period. There are three follow-up stages: 1, 2 and 4 months after randomisation. All follow-ups will be initiated by sending text messages to participants with hyperlinks to questionnaires. The following additional attempts will be made to collect data:

1. A total of two text reminders will be sent 2 days apart to those who have not responded.
2. If there is no response to the mediator questions at the 1-month follow-up, then the questions will be sent in a text message and participants are asked to respond directly with a text.
3. If there is no response to the 2-month and 4-month follow-ups, then we will call participants to collect responses for the primary outcome measures only. A maximum of five call attempts will be made.

### Assignment of interventions

Randomisation will be fully computerised, and allocation will be done automatically. Block randomisation will be used to allocate participants equally among the 64 factor conditions with random block sizes of 64 and 128. Neither research personnel nor participants will be able to influence the allocation.

Research personnel will be blind to allocation throughout the trial, although there is risk for potential disclosure during follow-up through phone calls (see the Limitations). The factorial design will allow all participants to have access to the intervention (although different components), and they will not be made aware of the other available conditions and will therefore be blind to allocation.

### Patient and participant involvement statement

During the process of designing the intervention content, 24 students at Linköping university were individually interviewed about their views on lifestyle in general, behaviour change, and the use of digital technology to support change. These interviews informed the content of the intervention, as did our previous research findings from formative development processes and user evaluation of digital interventions among Swedish university students.^29 65–67^ Our previous research also informed decisions about burden of the intervention and time required to participate.

Participants in the trial will after the 4-month follow-up be asked if they wish to participate in a post-trial interview. The purpose of the interviews is to explore strategies used by participants to change their behaviour. We will randomly select participants each week among those who report interest, stratifying for age, gender, baseline risk factors and allocation to ensure that we cover a heterogeneous group with respect to these variables, with a target of 20–30 interviews.

Outcome measures used in the trial are informed by national guidelines in Sweden, as well as those set by the WHO. Also, the Swedish National Board of Health and Welfare^59^ have reported that research regarding multiple lifestyle behaviour change interventions is lacking.

## Analysis

Analyses will be done under the intention-to-treat principle including all randomised participants. Analyses will be done using available data and imputation. Imputation will be done using multiple imputations with chained equations.^68^ The implicit missing at random (MAR) assumption underlying this approach will be investigated by two attrition analyses: (1) if data is missing systematically then it may be the case that early responders (ie, those who answer without reminders) differ from non-responders, and in extension that late responders (ie, those who require several attempts) are more alike non-responders. Therefore, one attrition analysis will regress primary outcomes against number of attempts to collect follow-up before a response was recorded; (2) we will further explore the MAR assumption by investigating if responders and non-responders are different with respect to baseline characteristics.

Longitudinal data will be analysed using multilevel models with adaptive intercepts for participants and time by component interactions. Bayesian inference will be used to estimate the parameters of the models^30 31 69^ (see Sample Size for priors). For each condition by time coefficient, we will report the marginal posterior probability of effect, and the median will be used as a point estimate of the magnitude of the effect. We will also report on 50% and 95% compatibility intervals.

### Models

#### Objective 1: primary and secondary outcomes

Analyses of primary outcomes will be conducted among those fulfilling the respective criteria for inclusion at baseline, for example, weekly alcohol consumption will be analysed among those who reported having consumed 10/15 (female/male) or more units of alcohol the past week. BMI, candy/snacks and stress will be analysed among all participants, and number of cigarettes smoked weekly among baseline smokers.

Weekly alcohol consumption, frequency of heavy episodic drinking per month, weekly intake of candy and snacks, number of sugary drinks per week, and cigarettes smoked per week are all count variables that are likely skewed and over dispersed. Therefore, these outcomes will be analysed using negative binomial regression. If found not to be over dispersed, we will consider using normal regression (possibly log-transformed). Average intake of fruit and vegetables per day, MVPA minutes per week, BMI, and stress will be analysed using normal regression (possibly log-transformed). Point prevalence of smoking abstinence will be analysed using logistic regression.

All models will be adjusted for age, gender and mediators at baseline. We will investigate pairwise interactions among components. Effect modification will be explored in all models to assess if any of the baseline characteristics moderate the effects of the components of the intervention.

#### Objective 2: mediator outcomes

Mediators will be explored using a causal inference framework,^70–72^ using Bayesian inference to estimate the natural direct effect and natural indirect effect (as per the definitions of Pearl^72^). We will report on the posterior distributions of these two estimates, as well as the proportion of the total effect which is accounted for by the natural indirect effect. Four models will be created for each primary outcome measure, three which investigate the mediating factors on their own, and a fourth which incorporates all mediators at once. If any baseline characteristics were found to moderate the effect in the primary analysis, then additional mediator models will be created to include these as moderators.

#### Objective 3: interactions among lifestyle change

Outcome interactions, and determinants of such, will be investigated in an exploratory analysis. For instance, those who quit smoking may also be more likely to reduce their alcohol consumption, and this interaction may be moderated by baseline characteristics. In addition, we will investigate interactions between changes in stress and behaviour change. Models to detect such interactions will be explored and findings will be used to create hypotheses for future research.

### Sample size, effect, harm and futility

The trial will use a Bayesian group sequential design^48–50^ to monitor recruitment with interim analyses planned for every 50 participants completing the 4-month follow-up. Each of the primary outcomes will be modelled according to the analysis plan (see Analysis), and coefficients for dummy variables representing presence/absence of each component will be assessed for effect, harm and futility with respect to each outcome. We let ß_k, l, i_ represent the regression coefficient for component *k*, at time l, for outcome *i*, and D all the data currently accumulated, then the target criteria will be:

- **Effect (fruit/vegetable and physical activity):** p(ß_k, l, i_ > 0 | D)>97.5% and p(ß_k, l, i_ > 0.10 | D)>50%.
- **Harm (fruit/vegetable and physical activity):** p(ß_k, l, i_ < 0 | D)>97.5% and p(ß_k, l, i_ < -0.10 | D)>50%.
- **Effect (sugary drinks, alcohol and smoking):** p(ß_k, l, i_ < 0 | D)>97.5% and p(ß_k, l, i_ < -0.10 | D)>50%.
- **Harm (sugary drinks, alcohol and smoking):** p(ß_k, l, i_ > 0 | D)>97.5% and p(ß_k, l, i_ > 0.10 | D)>50%.
- **Futility (all outcomes):** p(−0.10 < ß_k, l, i_ < 0.10 | D)>95%.

Outcomes analysed using normal regression will be standardised when checking the above criteria. For the effect and harm criteria, we will use a standard normal prior for dummy covariates (mean=0, SD=1.0), and a slightly wider prior will be used for the futility criterion (mean=0, SD=2.0). The criteria should be viewed as targets, thus, at each interim analysis, we will evaluate each criterion and make a decision if we believe that recruitment should stop or continue. We will consider removing factors from the trial if the harm criteria are fulfilled. Note that we are estimating each component’s effect on each outcome, thus we are not a priori excluding any combination. If a component is ineffective with respect to a specific outcome, then this will be captured by the futility criteria, and will also be reported as a finding.

While the final sample size is not determined a priori, we conducted a series of simulations with effect sizes at the minimal value of the above criteria (0.1 Cohen’s d for fruit/vegetable and physical activity, 1.1 incidence rate ratios for sugary drinks and alcohol and 1.1 ORs for smoking). Simulations suggested that approximately 1500–2500 participants will be necessary to recruit. However, the criteria will decide, not the simulations. Recruitment will last no longer than 24 months despite criteria being fulfilled or not. Despite having more conditions than in a traditional two-arm trial (in this case, 64 conditions), the factorial design is fully powered for each contrast.^47^ This can be understood by observing that half the study population are given access to each individual component (see Table 1 in online supplemental appendix 3), thus the other half creates a contrast (a type of control).

Note that the Bayesian approach allows us to make unlimited looks at the data without worrying about multiplicities and error rates, as would be necessary using a frequentist approach.^73^ Also, since no fixed effect size is prespecified, we reduce the risk of stopping recruitment both too early and too late.^50^

## Discussion

Many Swedish college and university students have unhealthy lifestyle behaviours, and trials of digital interventions have shown promising results with respect to behaviour change. Digital multiple lifestyle interventions have previously been investigated among other target populations, including both adolescent^74^ and adult populations,^75^ and non-digital multiple lifestyle interventions have been investigated in student settings.^76^ However, there is a paucity of studies of digital multiple behaviour change interventions targeting college and university students,^77^ thus, this study will add novel findings to a growing body of evidence.

In addition, behaviour interventions have predominantly been evaluated as a whole,^45^ which makes it uncertain *what works* within the intervention. A strength of the design of the trial described herein is that we will estimate the effects of the intervention components on the individual lifestyle behaviours, thus if there are components that seem ineffective, they can be further developed or removed. Factorial trials are not new;^47^ however, their use in mHealth is arguably underused.^45^ Other designs to evaluate components exist, which we anticipate will also become more common as the field progresses, including micro-randomisation and SMART trials.^78^

### Generalisability

Recruitment to this trial is done pragmatically through channels from which students would normally be approached with information about health and other services. Our inclusion criteria are not strongly prohibitive, rather, participants can be described as a population having at least one lifestyle behaviour which puts their physical and mental health at risk. Not all university students are at risk; however, it is unlikely that students who have healthy behaviours would seek help from this type of intervention if it was generally available. This strengthens the argument that the effects estimated in this trial are representative of what we could expect in a real-world implementation.

These generalisation arguments should be attenuated considering the fact that individuals who decide to take part in trials may be systematically different from those who do not. Also, being part of a trial may in and of itself change behaviour.^79 80^ We will not be able to assess these differences in this trial; thus, our findings should be viewed in light of potential (but unknown) systematic differences among those who are take part in this trial and those who would use the support in a real-world setting.

### Limitations

Lack of blinding is a high risk source of bias in trials,^81 82^ in particular, when outcomes are self-reported. Social desirability may be strong in such trials, and if the intervention group is positive to the treatment received, they may want to support its dissemination by reporting more positive outcomes than actual.^83^ Likewise, compensatory rivalry among participants who feel that they did not receive support which suited their needs may also bias results.^84^ The factorial design that we have chosen for this trial goes some way towards blinding participants to allocation (and awareness of other conditions). We are offering an intervention to all participants, and they are only told that they will receive one version of many. To investigate the validity of these assumptions, we will ask questions regarding participants’ perceptions about the support received. If there are patterns indicating that participants in any factorial condition(s) found that they did not receive adequate support, or if some are more positive than others to the support received, then our attempt to use the factorial design to blind participants in order to reduce these biases may not have succeeded.

A related concern is that condition allocation may be revealed when participants are called to collect follow-up data. Non-blinded assessment of subjective measures has in some studies found to bias estimates.^85^ While research personnel will be instructed not to ask or prompt participants to reveal allocation, it is likely that some participants may discuss the support they received. It is, however, unlikely that research personnel will be able to figure out the exact condition which participants have been allocated to, yet this does not completely remove the risk of detection bias. We do, however, believe that using phone calls to collect data at follow-up reduced the risk of attrition bias to such a degree that it outweighs the potential risk of detection bias, and the research personnel making the phone calls have experience of these types of studies and understand the risks involved.

Finally, we would like to address three methodological compromises. First, the use of single face-valid items for mediators. While it would be advantageous to use validated questionnaires for these factors, the participant burden would increase significantly, and we would risk high attrition. This means that any marked mediation will have to be carefully connected to the proposed considered factors, as the single items cannot capture the full concept of importance, confidence and know-how. Second, our criteria for stopping the trial are all based on the analysis of individual components and do not consider two-way interactions among components. Although it would be advantageous to power the study for these from a methodological standpoint, it is not practical to do so as it would increase the expected sample size markedly. Third, it is possible that participants have contact with each other, which means that there may be some cross-contamination. We decided against cluster randomisation as there would not be enough colleges or universities to assign the 64 conditions, and since there is no other unit of randomisation which would adequately shield against cross-contamination. Therefore, we accept that cross-contamination may bias estimates towards the null.

## Ethics and dissemination

The study was approved by the Swedish Ethical Review Authority on 2020-12-15 (Dnr 2020-05496). The main concern is the opportunity cost if the intervention is found to only have small effects. While participation in the study may partially be motivated by altruism, it is likely that most participants sign up motivated by the potential of support for behaviour change. In case the intervention is found to have very small effects, participants may have been more helped by engaging in other support during the 4-month study period and may be demotivated by not being successful. However, considering the lack of a generally available evidence-based multiple lifestyle behaviour support to university students, this risk was deemed acceptable given the potential benefits from the study.

Recruitment will begin in March 2021, and we anticipate that recruitment will last no more than 24 months. A final data set will therefore be available in July 2023, and findings will be submitted for peer review in open access journals no later than December 2023.
