# Supplementary material for: Multiple lifestyle behaviour mHealth intervention targeting Swedish college and university students: protocol for the Buddy randomised factorial trial
Source: BMJ Open. 2021 Dec 30;11(12):e051044. doi: 10.1136/bmjopen-2021-051044 (PMC8719203; doi:10.1136/bmjopen-2021-051044)
Supplement: Supplementary data [file bmjopen-2021-051044supp001.pdf]

APPENDIX A – INFORMED CONSENT

We would like to ask you to participate in a research project. On this page you can find information about the project and what participation entails.

WHY ARE WE DOING THIS STUDY AND WHY DO YOU WANT ME TO PARTICIPATE?

"BUDDY the Health Intervention" is a research project which aims to evaluate the effects of a digital support tool which has been designed to help college and university students to change their lifestyle behaviours. The support tool, which we call *BUDDY*, is delivered entirely through your mobile phone. Since you are a college or university student in Sweden, we would like to ask you if you would like to participate in the study.

The research project is conducted at Linköping University in Sweden, please find contact details for the primary investigator below.

WHAT IS EXPECTED OF ME?

Those who wish to participate in the study will first send a text message to a dedicate telephone number. A reply will be sent with a link to this page, which contains information about the study. Those who consent to take part in the study will then be asked to complete a short baseline questionnaire about their current lifestyle behaviours. As BUDDY is designed to help students change their alcohol, physical activity, diet and smoking behaviours, the baseline questionnaire will contain questions about these. After the baseline questionnaire has been completed, those who have at least one unhealthy lifestyle behaviour will be given access to BUDDY for four months. A text will be sent to participants immediately after completing the baseline questionnaire with information about how BUDDY works. As BUDDY is delivered through text messages, participants will not have to download any apps or do anything else than respond to the baseline questionnaire to get going.

BUDDY is a digital support tool which is designed to help students improve their lifestyle behaviours. There are several different versions of BUDDY, and which version you will be given access to will be decided randomly. BUDDY will support you by sending texts to you over a 4-month period. In the texts, there will be a link to your personalised support. The support is different depending on which version of BUDDY you have been assigned, and it may contain feedback, tips and tools, reflection, strategy building, support to build mental and physical resources, recommendations of other support available, and information about what is considered healthy lifestyle behaviours.

One, two and four months after BUDDY has started, all participants will be asked to complete follow-up questionnaires regarding their current lifestyle behaviours (on their phone via text messages with hyperlinks to questionnaires.). The questionnaires take 5-10 minutes to complete. In the final 4-month questionnaire, participants will be asked if they wish to take part in a telephone interview to talk about their experience of using BUDDY. Participation is complete after four months for those who do not want to take part in the interviews. Those wish to take part in the interviews will be contacted by phone.

WILL I BE TAKING ANY RISKS BY PARTICIPATING IN THIS STUDY?

If you decide to take part in the study then you should be aware that while BUDDY has been designed based on current scientific evidence regarding how to support individuals to change their behaviours, not everyone who uses BUDDY will succeed. This may feel like a failure and may be de-motivating. Participants should also be aware that changing lifestyle behaviours may result in discomfort, for instance withdrawal symptoms from

alcohol or nicotine. These discomforts are passing, and in the long run the health benefits outweigh these discomforts.

You can always decide to stop BUDDY, and we will not ask you why. Information about how to stop BUDDY will be given to you if you decide to participate. You can always contact you student or primary healthcare centre if you feel that you want more help with your health, or if you feel any discomforts. You can also contact 1177 if you have questions about lifestyle behaviours and health.

WILL I BE LEAVING ANY PERSONAL INFORMATION?

The project will collect information about you.

Your responses to the questionnaires during the study period will be stored in a database at Linköping university. We will use an encrypted version of your phone number in order to connect your responses from the different questionnaires. The secret key used to decrypt the phone number will only be accessible by the primary investigator Marcus Bendtsen (see contact information below). We will also collect information about how you use BUDDY, so that we can analyse usage of the support tool. Only researchers at Linköping university who are part of the research project will have access to the data collected in this study. Neither during analysis nor publication of findings will any information be connected to you individually.

When the project is complete, all phone numbers will be deleted, and the collected data will then become anonymous. The anonymous information will be stored securely at Linköpings University for 10 years.

The data collection is for scientific research and is therefore motivated by public interest (GDPR EU 2016/679, Prop. 2017/18:298).

Your data will be stored so that only authorised researchers have access to them. Linköping University are responsible for your data. According to EU:s data protection regulation you have the right to, free of charge, be given access to the data collected about you in the project, and have errors corrected. You may also request that your data be deleted or use of your data be restricted. If you want to be given access to your data, you should contact the primary investigator Marcus Bendtsen (see contact information below). The data protection officer can be reached at dataskyddsombud@liu.se. If you are not satisfied with the way your personal data has been handled, you can file a complaint at the Swedish Data Protection Authority.

HOW CAN I GET MORE INFORMATION ABOUT THE RESULTS FROM THIS STUDY?

After the study has been completed, findings will be published in scientific peer-reviewed journals. Findings at the individual level will not be traceable from these publications. We will not contact you after study completion, but you are welcome to contact us if you would like copies of published reports.

INSURANCE

As a participant of a research project at Linköping University you are included in the insurance the university has at Kammarkollegiet.

PARTICIPATION IS FREE

Participation is free and you can at any time decide to end it. If you decide to end your participation you will not be asked why, and it will not affect future care, treatment or your studies. If you wish to end your participation you should contact the primary investigator Marcus Bendtsen (see below).

HOW WILL THE SUPPORT BE DISSEMINATED?

If this project finds that BUDDY has a positive effect on students' behaviours, then it will be made freely available to Swedish students. The primary investigator Marcus Bendtsen owns a company (Alexit AB) which may be responsible for the dissemination. Alexit AB does not have access to any data from this study, and has no influence on the research questions or analyses.

PRIMARY INVESTIGATOR

Marcus Bendtsen, PhD
Docent in Medical Informatics
Senior Lecturer in Experimental Social Medicine and Public Health
Department of Health Medicine and Caring Sciences, Linköping University
marcus.bendtsen@liu.se, 013-286975
