# Supplementary material for: Multiple lifestyle behaviour mHealth intervention targeting Swedish college and university students: protocol for the Buddy randomised factorial trial
Source: BMJ Open. 2021 Dec 30;11(12):e051044. doi: 10.1136/bmjopen-2021-051044 (PMC8719203; doi:10.1136/bmjopen-2021-051044)
Supplement: Supplementary data [file bmjopen-2021-051044supp002.pdf]

APPENDIX B – QUESTIONNAIRES

BASELINE QUESTIONNAIRE

1. Sex:
    a. Female
    b. Male

2. Age (numerical measure)

3. How many standard drinks of alcohol did you consume last week? (numerical measure)

4. How often, during the past month, have you consumed four/five (female/male) or more standard drinks of alcohol on one occasion? (numerical measure)

5. How many cigarettes did you smoke last week? (numerical measure)

6. How much time in total did you spend on moderate physical activity (e.g. bicycling or walking for transport or leisure) **last week**?
    a. 0
    b. Less than 30 minutes
    c. 30-60 minutes
    d. 1 hours
    e. 1.5 hours
    f. 2 hours
    g. 2.5 hours
    h. 3 hours
    i. 3.5 hours (i.e. 30 minutes per day)
    j. 4 hours
    k. 5 hours
    l. 6 hours
    m. 7 hours (i.e. 1 hour per day)
    n. 10.5 hours (i.e. 1.5 hours per day)
    o. 14 hours (i.e. 2 hours per day)

7. How much time in total did you spend on vigorous physical activity (i.e. producing fast increases in breathing or heart rate), for instance running, aerobics, etc. **last week?**
    a. 0
    b. Less than 30 minutes
    c. 30-60 minutes
    d. 1 hours
    e. 1.5 hours
    f. 2 hours
    g. 2.5 hours
    h. 3 hours
    i. 3.5 hours (i.e. 30 minutes per day)
    j. 4 hours
    k. 5 hours

l. 6 hours
m. 7 hours (i.e. 1 hour per day)
n. 10.5 hours (i.e. 1.5 hours per day)
o. 14 hours (i.e. 2 hours per day)

8. How many 100g portions (equivalent to an average sized banana or one large apple) of fruit did you consume **last week**?
   a. 0
   b. 1-2 portions **per week**
   c. 3-4 portions **per week**
   d. 5-6 portion **per week**
   e. 1.0 portion **per day**
   f. 1.5 portions **per day**
   g. 2.0 portions **per day**
   h. 2.5 portions **per day**
   i. 3.0 portions **per day or more**

9. How many 100 g portions (equivalent to an average handful) of vegetables did you consume **last week**?
   a. 0
   b. 1-2 portions **per week**
   c. 3-4 portions **per week**
   d. 5-6 portion **per week**
   e. 1.0 portion **per day**
   f. 1.5 portions **per day**
   g. 2.0 portions **per day**
   h. 2.5 portions **per day**
   i. 3.0 portions **per day or more**

10. How many cans (33 cl, one standard can) of sugary drinks (e.g. soft/fizzy drinks, “energy drinks”) did you consume **last week**?
   a. 0 cans
   b. 1 can **per week**
   c. 2-3 cans **per week**
   d. 4-6 cans **per week**
   e. 1 can **per day**
   f. 1.5 cans **per day**
   g. 2.0 cans **per day**
   h. 2.5 cans **per day**
   i. 3.0 cans **per day or more**

11. How many portions of sweets, chocolate, pastry (e.g. buns, muffins, biscuits), ice cream and salty snacks (e.g. crisps, nuts, cheese doodles) did you eat **last week**? *One portion is 50 g sweets (9 pieces), 40 g chocolate (6 pieces/squares), 1 bun, 2 dl (scoops) of ice cream or 2 dl snacks (40 g)*.

   a. 0 portions
   b. 1 portion **per week**
   c. 2-3 portions **per week**
   d. 4-6 portions **per week**
   e. 1 portion **per day**

- **f.** 1.5 portions **per day**
- g. 2.0 portions **per day**
- h. 2.5 portions **per day**
- i. 3.0 portions **per day**
- j. 3.5 portions **per day**
- k. 4.0 portions **per day or more**

12. How tall are you? (numerical measure)

13. What is your current body weight? (numerical measure)

14. In the last month, how often have you felt that you were unable to control the important things in your life?
    a. Never
    b. Almost never
    c. Sometimes
    d. Fairly often
    e. Very often

15. In the last month, how often have you felt confident about your ability to handle your personal problems?
    a. Never
    b. Almost never
    c. Sometimes
    d. Fairly often
    e. Very often

16. In the last month, how often have you felt that things were going your way?
    a. Never
    b. Almost never
    c. Sometimes
    d. Fairly often
    e. Very often

17. In the last month, how often have you felt difficulties were piling up so high that you could not overcome them?
    a. Never
    b. Almost never
    c. Sometimes
    d. Fairly often
    e. Very often

18. How important is it for you to improve your lifestyle behaviours? (10-point scale ranging from 1 = “Not important” to 10 = “Very important”)

19. How confident are you that you will be able to improve your lifestyle behaviours? (10-point scale ranging from 1 = “Not at all” to 10 = “Very confident”)

20. To what degree do you have the know-how and strategies to improve your lifestyle behaviours? (10-point scale ranging from 1 = “Not at all” to 10 = “Very high degree”)

Note: Participants are reminded of the definition of a standard unit of alcohol by graphical means, as well as given visual cues for what constitutes a portion of fruit, vegetables and a unit of sugary drinks.

1-MONTH FOLLOW-UP (MEDIATORS ONLY) QUESTIONNAIRE

1. How important is it for you to improve your lifestyle behaviours? (10-point scale ranging from 1 = "Not important" to 10 = "Very important")
2. How confident are you that you will be able to improve your lifestyle behaviours? (10-point scale ranging from 1 = "Not at all" to 10 = "Very confident")
3. To what degree do you have the know-how and strategies to improve your lifestyle behaviours? (10-point scale ranging from 1 = "Not at all" to 10 = "Very high degree")

2- AND 4-MONTH FOLLOW-UP QUESTIONNAIRE

1. How many standard drinks of alcohol did you consume last week? (numerical measure)
2. How often, during the past month, have you consumed four/five (female/male) or more standard drinks of alcohol on one occasion? (numerical measure)
3. Have you smoked any cigarettes the past four weeks?
   a. Yes
   b. No
4. (Smokers only) How many cigarettes did you smoke last week? (numerical measure)
5. How much time in total did you spend on moderate physical activity (e.g. bicycling or walking for transport or leisure) **last week**?
   a. 0
   b. Less than 30 minutes
   c. 30-60 minutes
   d. 1 hours
   e. 1.5 hours
   f. 2 hours
   g. 2.5 hours
   h. 3 hours
   i. 3.5 hours (i.e. 30 minutes per day)
   j. 4 hours
   k. 5 hours
   l. 6 hours
   m. 7 hours (i.e. 1 hour per day)
   n. 10.5 hours (i.e. 1.5 hours per day)
   o. 14 hours (i.e. 2 hours per day)
6. How much time in total did you spend on vigorous physical activity (i.e. producing fast increases in breathing or heart rate), for instance running, aerobics, etc. **last week?**
   a. 0
   b. Less than 30 minutes
   c. 30-60 minutes

d. 1 hours
e. 1.5 hours
f. 2 hours
g. 2.5 hours
h. 3 hours
i. 3.5 hours (i.e. 30 minutes per day)
j. 4 hours
k. 5 hours
l. 6 hours
m. 7 hours (i.e. 1 hour per day)
n. 10.5 hours (i.e. 1.5 hours per day)
o. 14 hours (i.e. 2 hours per day)

7. How many 100g portions (equivalent to an average sized banana or one large apple) of fruit did you consume **last week**?
   a. 0
   b. 1-2 portions **per week**
   c. 3-4 portions **per week**
   d. 5-6 portion **per week**
   e. 1.0 portion **per day**
   f. 1.5 portions **per day**
   g. 2.0 portions **per day**
   h. 2.5 portions **per day**
   i. 3.0 portions **per day or more**

8. How many 100 g portions (equivalent to an average handful) of vegetables did you consume **last week**?
   a. 0
   b. 1-2 portions **per week**
   c. 3-4 portions **per week**
   d. 5-6 portion **per week**
   e. 1.0 portion **per day**
   f. 1.5 portions **per day**
   g. 2.0 portions **per day**
   h. 2.5 portions **per day**
   i. 3.0 portions **per day or more**

9. How many cans (33 cl, one standard can) of sugary drinks (e.g. soft/fizzy drinks, “energy drinks”) did you consume **last week**?
   a. 0 cans
   b. 1 can **per week**
   c. 2-3 cans **per week**
   d. 4-6 cans **per week**
   e. 1 can **per day**
   f. 1.5 cans **per day**
   g. 2.0 cans **per day**
   h. 2.5 cans **per day**
   i. 3.0 cans **per day or more**

10. How many portions of sweets, chocolate, pastry (e.g. buns, muffins, biscuits), ice cream and salty snacks (e.g. crisps, nuts, cheese doodles) did you eat **last week**? *One portion is 50 g sweets (9 pieces), 40 g chocolate (6 pieces/squares), 1 bun, 2 dl (scoops) of ice cream or 2 dl snacks (40 g)*.

    a. 0 portions
    b. 1 portion **per week**
    c. 2-3 portions **per week**
    d. 4-6 portions **per week**
    e. 1 portion **per day**
    f. 1.5 portions **per day**
    g. 2.0 portions **per day**
    h. 2.5 portions **per day**
    i. 3.0 portions **per day**
    j. 3.5 portions **per day**
    k. 4.0 portions **per day or more**

11. What is your current body weight? (numerical measure)

12. In the last month, how often have you felt that you were unable to control the important things in your life?
    a. Never
    b. Almost never
    c. Sometimes
    d. Fairly often
    e. Very often

13. In the last month, how often have you felt confident about your ability to handle your personal problems?
    a. Never
    b. Almost never
    c. Sometimes
    d. Fairly often
    e. Very often

14. In the last month, how often have you felt that things were going your way?
    a. Never
    b. Almost never
    c. Sometimes
    d. Fairly often
    e. Very often

15. In the last month, how often have you felt difficulties were piling up so high that you could not overcome them?
    a. Never
    b. Almost never
    c. Sometimes
    d. Fairly often
    e. Very often

16. How important is it for you to improve or maintain healthy lifestyle behaviours? (10-point scale ranging from 1 = “Not important” to 10 = “Very important”)

17. How confident are you that you will be able to improve or maintain healthy lifestyle behaviours? (10-point scale ranging from 1 = “Not at all” to 10 = “Very confident”)

18. To what degree do you have the know-how and strategies to improve or maintain healthy lifestyle behaviours? (10-point scale ranging from 1 = “Not at all” to 10 = “Very high degree”)

4-MONTH FOLLOW-UP ONLY (PERCEIVED USEFULNESS AND GENERAL OPINION OF THE SUPPORT RECEIVED)

1. Would you like to participate in a telephone-interview to discuss how you used and reacted to the support you received?
    a. Yes, I would like to participate, and it is OK if you call me to setup a meeting.
    b. No.

2. Overall, how well do you believe that the support given to you suited your needs?
    a. I feel like I did not receive any support at all
    b. I feel like I received some support, but it did not suit my needs
    c. I feel like I received some support, and it did suit my needs
    d. I feel like I received all the support that I needed

3. (If a or b to question 2): You have responded that you did not receive adequate support, what did you do instead?
    a. I decided to find other ways to help me change my lifestyle
    b. I decided to not make any change to my lifestyle
    c. Other (please comment)

4. Please leave a comment describing your needs and how the support did or did not address them (Free-text).

5. Do you believe that the support given to you would be helpful for other individuals that want to change their lifestyle? (1 = “Not very helpful” to 5 = “Very helpful”)

6. Would you recommend the support you were given to a friend who expresses a wish to change their lifestyle?
    a. Yes
    b. No
    c. I do not know

7. If you were to continue using the support, for how much longer would you want to use it?
    a. I would use it for one to two more months
    b. I would use it for three to six more months
    c. I would use it for more than six months
    d. I would not use it any more
    e. I do not know
