# Supplementary material for: Multiple lifestyle behaviour mHealth intervention targeting Swedish college and university students: protocol for the Buddy randomised factorial trial
Source: BMJ Open. 2021 Dec 30;11(12):e051044. doi: 10.1136/bmjopen-2021-051044 (PMC8719203; doi:10.1136/bmjopen-2021-051044)
Supplement: Supplementary data [file bmjopen-2021-051044supp003.pdf]

APPENDIX C – INTERVENTION DESCRIPTION AND FACTORIAL CONDITIONS

This appendix describes the development and the content of the BUDDY intervention, with inspiration from the first four steps of the Intervention mapping (IM) approach [1]. The intervention targets alcohol, diet, physical activity, and smoking. The factorial conditions of the trial are also explained in detail in this appendix.

The intervention is based on social cognitive models for behaviour change where environment, intentions and skills often are highlighted as important for change [2,3]. Therefore, we identified and designed components which intended to affect these factors. This was based on: (1) our previous research among Swedish university students [4–10]; (2) a series of interviews with university students regarding their perspectives on health, lifestyle behaviour change, and expectations on an mHealth tool supporting behaviour change; (3) the research literature more widely (see specific descriptions below). The logic model in Figure 1 gives an overview of the reasoning behind the intervention, including outcomes and potential short-, mid- and long-term impact.

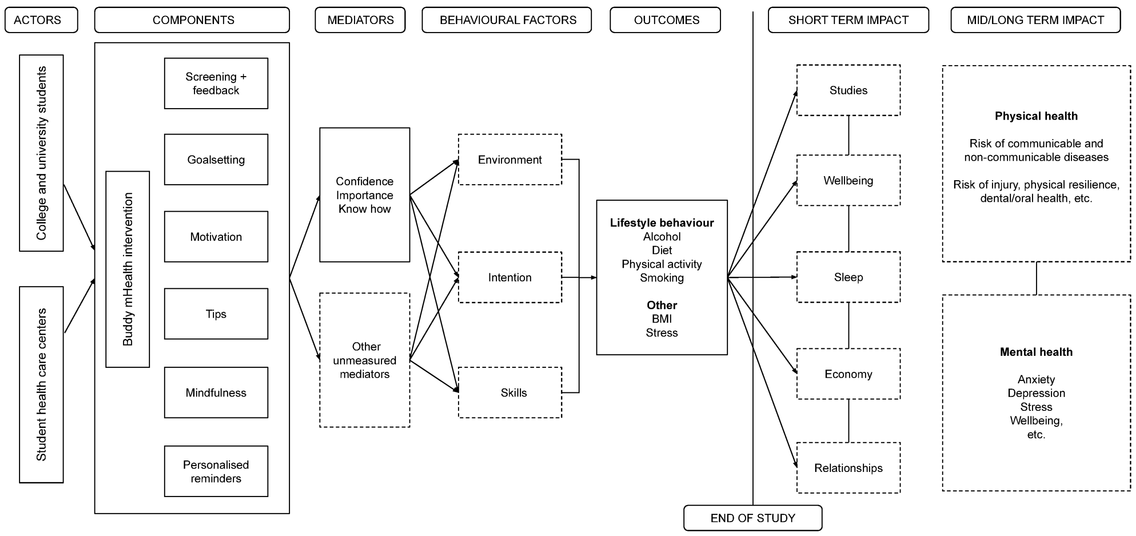

Figure 1. Logic model showing actors, intervention components, mediators, behavioural factors, outcomes, and short-, mid- and long-term impacts

INTERVENTION DESIGN AND COMPONENTS

The intervention is intended to be used as a toolbox, enabling users to decide which intervention content they want to interact with and when. As can be seen in the screenshot in Figure 2, the design of the intervention allowed for each component to be presented to participants in a menu, allowing us to easily add or remove components based on factorial condition. Follows does a description of each component, using the BCTTv1 93-item taxonomy [11] to specify techniques included when appropriate.

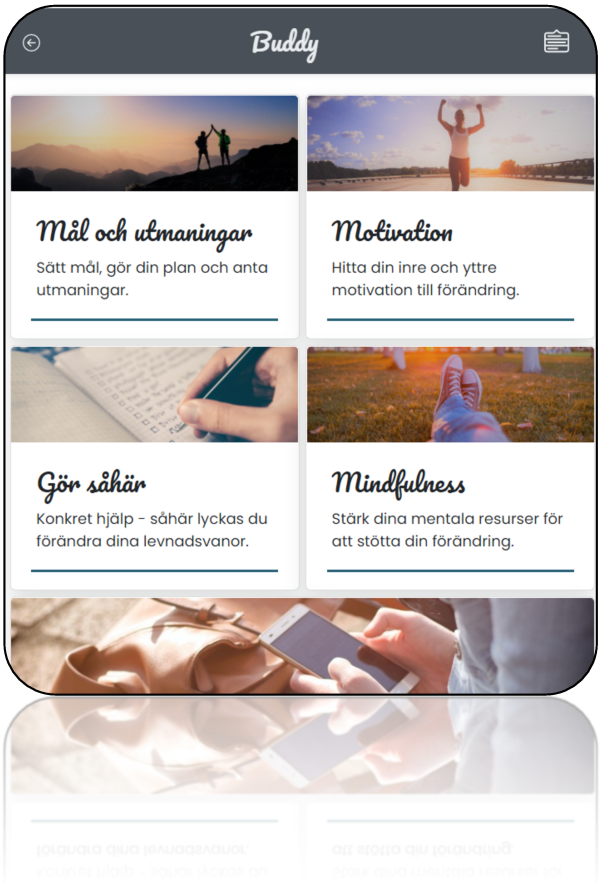

Figure 2. A screenshot of the BUDDY intervention showing the main menu

COMPONENT 1: SCREENING AND FEEDBACK

The first component consists of screening and feedback. Every Sunday afternoon, participants will receive a text message with a hyperlink. When pressing the link, participants will be asked to respond to a questionnaire regarding their current lifestyle behaviours, after which they are shown feedback on their current behaviour in contrast to national guidelines (see screenshots in Figure 3). They will subsequently be given access to the rest of the components appropriate for their randomised allocation. Self-monitoring has been shown to be a potentially effective strategy for reducing excessive alcohol consumption [12–15] and to promote healthy eating and physical activity [16,17]. When this component is absent, participants will not be asked to respond to the screening questionnaire but will instead be shown national guidelines without any feedback. BCTs used: Discrepancy between current behaviour and goal (BCT 1.6), Feedback on behaviour (BCT 2.2), Self-monitoring of behaviour (BCT 2.3), and Social comparison (BCT 6.2).

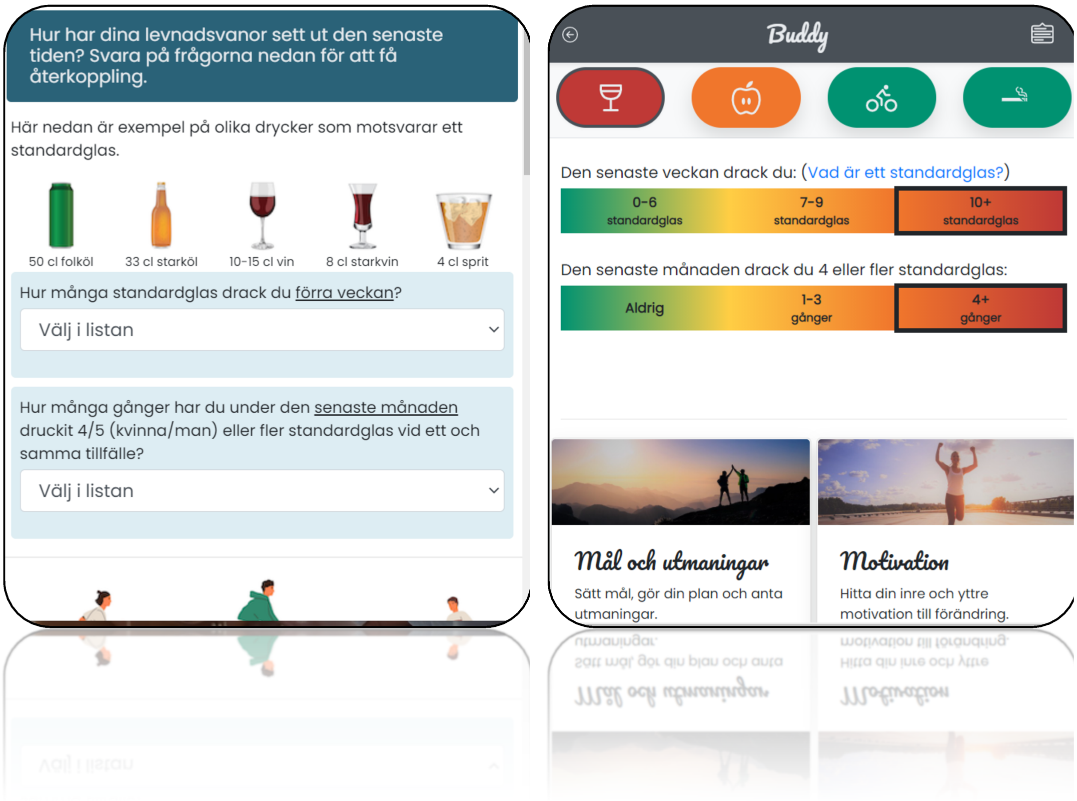

Figure 2 – Screenshots of BUDDY showing screening and feedback based on national guidelines

COMPONENT 2: GOALSETTING AND PLANNING

The second component supports enhanced self-regulatory capacity and skills via goalsetting and planning. This includes setting goals for future behaviour, preparing for triggers, and accepting both custom and ready-made challenges. Intervention content designed around goalsetting, action planning, practicing behaviour, and habit formation have, amongst other planning related activities, been shown to be important among effective lifestyle interventions [16,18–23]. Participants will be reminded of the goals that they have set, including any challenges they have accepted, via text message prompts throughout the week (up to 4 messages). BCTs used: Goal setting (behaviour) (BCT 1.1), Problem solving (BCT 1.2), Action planning (BCT 1.4), Prompts/cues (BCT 7.1), Behaviour practice/rehearsal (BCT 8.1), Behaviour substitution (BCT 8.2), Habit formation (BCT 8.3), Graded tasks (BCT 8.7).

COMPONENT 3: MOTIVATION

The third component aims to increase users' awareness of their own motivation, prompt commitment, and boost motivation. This is supported via texts, videos and exercises relating to health, economics, and motivation awareness. Digital behaviour change interventions have been shown to have the capacity to increase self-efficacy, however, there is lack of consensus across reviews with regards to which content works to facilitate an increase of self-efficacy [24]. The component will also allow participants to sign up for text

messages with motivational content sent to them throughout the week. Participants choose which behaviours they wish to have messages for, with a maximum of 8-10 messages per week. The content of the messages has been derived from previously developed and evaluated interventions [4–10]. BCTs used: Information about health consequences (BCT 5.1), Credible source (9.1), Pros and cons (BCT 9.2), Comparative imagining of future outcomes (BCT 9.3).

COMPONENT 4: SKILLS AND KNOW-HOW

The fourth component aims to increase user's skills and know-how of how to make lasting behavioural changes. This will include concrete tips on how to initiate and maintain change in everyday life. For instance, participants are given strategies they can employ when going to parties where alcohol is served, or how to introduce vegetables to their meals. As with the third component, participants will be able to sign up for text messages with tips sent to them throughout the week (maximum 8-10 per week) – the content of which has also been derived from previously developed and evaluated interventions [4–10]. BCTs used: Social support (unspecified) (BCT 3.1), Instructions on how to perform a behaviour (BCT 4.1), Self-incentive (BCT 10.7), and Self-reward (BCT 10.9).

COMPONENT 5: MINDFULNESS

The fifth component aims to increase users' awareness of their own lived experience and strengthen their capacity for a non-reactive, compassionate, and less stressful way of being in the world. The practices thus help participants to build the mental resources needed for behaviour change. A set of mindfulness exercises, including guided meditations, will be available in the component. The exercises are based on previous research, and are considered evidence-based methods to improve the mental well-being of clinical populations, while effects in non-clinical settings and behaviour change are less studied [25–29].

COMPONENT 6: SELF-COMPOSED TEXT MESSAGES

The sixth component consists of self-composed text messages sent to participants throughout the week. Participants will be allowed to author up to three messages to themselves and have them sent at specified intervals. For instance, a participant can write a message about their commitment to increase their physical activity and decide to have it sent to them every Monday and Wednesday at 5pm. This type of activity seems generally under-studied in the literature, but has shown preliminary interesting results in an ongoing trial [30].

FACTORIAL ALLOCATIONS

Using P to represent *present* and A to represent *absent*, the 64 factorial conditions are presented in Table 1. As is evident from the table, each component will be available to half the study population, allowing for contrasts between *present* and *absent* to be fully powered by the sample. However, as one is estimating the effects of individual components, effect sizes may be smaller than when contrasting the full intervention versus a control, which may increase the sample size required.

Table 1 - Combination of components in 64 factorial conditions (P = present , A = absent)

Conditions	Component 1	Component 2	Component 3	Component 4	Component 5	Component 6
1	P	P	P	P	P	P
2	P	P	P	P	P	A
3	P	P	P	P	A	P
4	P	P	P	P	A	A
5	P	P	P	A	P	P
6	P	P	P	A	P	A
7	P	P	P	A	A	P
8	P	P	P	A	A	A
9	P	P	A	P	P	P
10	P	P	A	P	P	A
11	P	P	A	P	A	P
12	P	P	A	P	A	A
13	P	P	A	A	P	P
14	P	P	A	A	P	A
15	P	P	A	A	A	P
16	P	P	A	A	A	A
17	P	A	P	P	P	P
18	P	A	P	P	P	A
19	P	A	P	P	A	P
20	P	A	P	P	A	A
21	P	A	P	A	P	P
22	P	A	P	A	P	A
23	P	A	P	A	A	P
24	P	A	P	A	A	A
25	P	A	A	P	P	P
26	P	A	A	P	P	A
27	P	A	A	P	A	P
28	P	A	A	P	A	A
29	P	A	A	A	P	P
30	P	A	A	A	P	A
31	P	A	A	A	A	P
32	P	A	A	A	A	A
33	A	P	P	P	P	P

34	A	P	P	P	P	A
35	A	P	P	P	A	P
36	A	P	P	P	A	A
37	A	P	P	A	P	P
38	A	P	P	A	P	A
39	A	P	P	A	A	P
40	A	P	P	A	A	A
41	A	P	A	P	P	P
42	A	P	A	P	P	A
43	A	P	A	P	A	P
44	A	P	A	P	A	A
45	A	P	A	A	P	P
46	A	P	A	A	P	A
47	A	P	A	A	A	P
48	A	P	A	A	A	A
49	A	A	P	P	P	P
50	A	A	P	P	P	A
51	A	A	P	P	A	P
52	A	A	P	P	A	A
53	A	A	P	A	P	P
54	A	A	P	A	P	A
55	A	A	P	A	A	P
56	A	A	P	A	A	A
57	A	A	A	P	P	P
58	A	A	A	P	P	A
59	A	A	A	P	A	P
60	A	A	A	P	A	A
61	A	A	A	A	P	P
62	A	A	A	A	P	A
63	A	A	A	A	A	P
64	A	A	A	A	A	A

REFERENCES

1. Bartholomew Eldredge LK. Planning health promotion programs: an intervention mapping approach. Fourth edition. San Francisco, CA: Jossey-Bass & Pfeiffer Imprints, Wiley; 2016. 1 p.

2. Fishbein M, Triandis HC, Kanfer FH, Becker M, Middlestadt SE, Eichler A. Factors influencing behaviour and behaviour change. In: Handbook of Health Psychology. Psychology Press Taylor & Francis Group; 2001. p. 3–17.

3. Conner M, Norman P. Predicting health behaviour: research and practice with social cognition models [Internet]. Open University Press; 2005. Available from: https://books.google.se/books?id=MZhzQgAACAAJ

4. Müssener U, Bendtsen M, Karlsson N, White IR, McCambridge J, Bendtsen P. Effectiveness of Short Message Service Text-Based Smoking Cessation Intervention Among University Students. JAMA Internal Medicine. 2016;176(3):321.

5. Müssener U, Bendtsen M, Karlsson N, White IR, McCambridge J, Bendtsen P. SMS-based smoking cessation intervention among university students: study protocol for a randomised controlled trial (NEXit trial). Trials. 2015;16(1):140.

6. Thomas K, Bendtsen M, Linderoth C, Karlsson N, Bendtsen P, Müssener U. Short message service (SMS)-based intervention targeting alcohol consumption among university students: study protocol of a randomized controlled trial. Trials. 2017;18(1):156.

7. Thomas K, Müssener U, Linderoth C, Karlsson N, Bendtsen P, Bendtsen M. Effectiveness of a Text Messaging–Based Intervention Targeting Alcohol Consumption Among University Students: Randomized Controlled Trial. JMIR mHealth and uHealth. 2018;6(6):e146.

8. Müssener U, Bendtsen M, McCambridge J, Bendtsen P. User satisfaction with the structure and content of the NEXit intervention, a text messaging-based smoking cessation programme. BMC Public Health. 2016;16(1):1179.

9. Mussener U, Thomas K, Linderoth C, Leijon M, Bendtsen M. A Text Message-Based Intervention Targeting Alcohol Consumption Among University Students: User Satisfaction and Acceptability Study. JMIR human factors. 2018;5(3):e23.

10. Thomas K, Linderoth C, Bendtsen M, Bendtsen P, Mussener U. Text Message-Based Intervention Targeting Alcohol Consumption Among University Students: Findings From a Formative Development Study. JMIR mHealth and uHealth. 2016;4(4):e119.

11. Michie S, Richardson M, Johnston M, Abraham C, Francis J, Hardeman W, et al. The Behavior Change Technique Taxonomy (v1) of 93 Hierarchically Clustered Techniques: Building an International Consensus for the Reporting of Behavior Change Interventions. Annals of Behavioral Medicine. 2013 Aug;46(1):81–95.

12. Michie S, Whittington C, Hamoudi Z, Zarnani F, Tober G, West R. Identification of behaviour change techniques to reduce excessive alcohol consumption. Addiction (Abingdon, England). 2012 Aug;107(8):1431–40.

13. Bendtsen P, McCambridge J, Bendtsen M, Karlsson N, Nilsen P. Effectiveness of a Proactive Mail-Based Alcohol Internet Intervention for University Students: Dismantling the Assessment and Feedback Components in a Randomized Controlled Trial. Journal of Medical Internet Research. 2012 Oct 31;14(5):e142.

14. McCambridge J, Bendtsen M, Karlsson N, White IR, Nilsen P, Bendtsen P. Alcohol assessment and feedback by email for university students: Main findings from a randomised controlled trial. British Journal of Psychiatry. 2013;203(5):334–40.

15. Bendtsen P, Bendtsen M, Karlsson N, White IR, McCambridge J. Online Alcohol Assessment and Feedback for Hazardous and Harmful Drinkers: Findings From the AMADEUS-2 Randomized Controlled Trial of Routine Practice in Swedish Universities. Journal of Medical Internet Research. 2015;17(7):e170.

16. Michie S, Abraham C, Whittington C, Mcateer J. Effective Techniques in Healthy Eating and Physical Activity Interventions : A Meta-Regression. 2009;28(6):690–701.

17. Murray JM, Brennan SF, French DP, Patterson CC, Kee F, Hunter RF. Effectiveness of physical activity interventions in achieving behaviour change maintenance in young and middle aged adults: A systematic review and meta-analysis. Social science & medicine (1982). 2017 Nov;192:125–33.

18. Knittle K, Nurmi J, Crutzen R, Hankonen N, Beattie M, Dombrowski SU. How can interventions increase motivation for physical activity? A systematic review and meta-analysis. Health psychology review. 2018 Sep;12(3):211–30.

19. Howlett N, Trivedi D, Troop NA, Chater AM. Are physical activity interventions for healthy inactive adults effective in promoting behavior change and maintenance, and which behavior change techniques are effective? A systematic review and meta-analysis. Translational behavioral medicine. 2019 Jan;9(1):147–57.

20. Ashton LM, Sharkey T, Whatnall MC, Williams RL, Bezzina A, Aguiar EJ, et al. Effectiveness of Interventions and Behaviour Change Techniques for Improving Dietary Intake in Young Adults: A Systematic Review and Meta-Analysis of RCTs. Nutrients. 2019 Apr;11(4).

21. Garnett C V, Crane D, Brown J, Kaner EFS, Beyer FR, Muirhead CR, et al. Behavior Change Techniques Used in Digital Behavior Change Interventions to Reduce Excessive Alcohol Consumption: A Meta-regression. Annals of behavioral medicine : a publication of the Society of Behavioral Medicine. 2018 May;52(6):530–43.

22. McCrabb S, Baker AL, Attia J, Skelton E, Twyman L, Palazzi K, et al. Internet-Based Programs Incorporating Behavior Change Techniques Are Associated With Increased Smoking Cessation in the General Population: A Systematic Review and Meta-analysis. Annals of behavioral medicine : a publication of the Society of Behavioral Medicine. 2019 Feb;53(2):180–95.

23. Ek A, Alexandrou C, Söderström E, Bergman P, Delisle Nyström C, Direito A, et al. Effectiveness of a 3-month mobile phone based behavior change program on active transportation and physical activity in adults: A randomized controlled trial. JMIR mHealth uHealth. 2020;8(6).

24. Newby K, Teah G, Cooke R, Li X, Brown K, Salisbury-Finch B, et al. Do automated digital health behaviour change interventions have a positive effect on self-efficacy? A systematic review and meta-analysis. Health Psychology Review. 2020 Jan 20;1–19.

25. Crane RS, Brewer J, Feldman C, Kabat-Zinn J, Santorelli S, Williams JMG, et al. What defines mindfulness-based programs? The warp and the weft. Psychological Medicine. 2017 Apr 29;47(6):990–9.

26. Creswell JD. Mindfulness Interventions. Annual Review of Psychology. 2017 Jan 3;68(1):491–516.

27. Wong SYS, Chan JYC, Zhang D, Lee EKP, Tsoi KKF. The Safety of Mindfulness-Based Interventions: a Systematic Review of Randomized Controlled Trials. Mindfulness. 2018 Oct 2;9(5):1344–57.

28. Galante J, Friedrich C, Dawson AF, Modrego-Alarcón M, Gebbing P, Delgado-Suárez I, et al. Mindfulness-based programmes for mental health promotion in adults in nonclinical settings: A systematic review and meta-analysis of randomised controlled trials. Patel V, editor. PLOS Medicine. 2021 Jan 11;18(1):e1003481.

29. Neff KD, Germer CK. A Pilot Study and Randomized Controlled Trial of the Mindful Self-Compassion Program. Journal of Clinical Psychology. 2013 Jan;69(1):28–44.

30. Bendtsen M, McCambridge J. Reducing Alcohol Consumption Among Risky Drinkers in the General Population of Sweden Using an Interactive Mobile Health Intervention: Protocol for a Randomized Controlled Trial. JMIR Research Protocols. 2019;8(4):e13119.
